# Viscosin-like lipopeptides from frog skin bacteria inhibit *Aspergillus fumigatus* and *Batrachochytrium dendrobatidis* detected by imaging mass spectrometry and molecular networking

**DOI:** 10.1038/s41598-019-39583-7

**Published:** 2019-02-28

**Authors:** Christian Martin H., Roberto Ibáñez, Louis-Félix Nothias, Cristopher A. Boya P., Laura K. Reinert, Louise A. Rollins-Smith, Pieter C. Dorrestein, Marcelino Gutiérrez

**Affiliations:** 1 Centro de Biodiversidad y Descubrimiento de Drogas, Instituto de Investigaciones Científicas y Servicios de Alta Tecnología (INDICASAT AIP), Panama, Republic of Panama; 2 Department of Biotechnology, Acharya Nagarjuna University, Guntur, India; 3 Smithsonian Tropical Research Institute, Balboa, Ancon Republic of Panama; 4 Departamento de Zoología, Universidad de Panamá, Panama, Republic of Panama; 5 Collaborative Mass Spectrometry Innovation Center, Skaggs School of Pharmacy and Pharmaceutical Sciences, University of California San Diego, La Jolla, California, USA; 6 Department of Pathology, Microbiology, and Immunology, and Department of Pediatrics, Vanderbilt University School of Medicine, Nashville, Tennessee USA; 7 Department of Biological Sciences, Vanderbilt University, Nashville, Tennessee USA

## Abstract

Amphibian populations worldwide have declined and in some cases become extinct due to chytridiomycosis, a pandemic disease caused by the fungus *Batrachochytrium dendrobatidis*; however, some species have survived these fungal epidemics. Previous studies have suggested that the resistance of these species is due to the presence of cutaneous bacteria producing antifungal metabolites. As our understanding of these metabolites is still limited, we assessed the potential of such compounds against human-relevant fungi such as *Aspergillus*. In this work we isolated 201 bacterial strains from fifteen samples belonging to seven frog species collected in the highlands of Panama and tested them against *Aspergillus fumigatus*. Among the 29 bacterial isolates that exhibited antifungal activity, *Pseudomonas cichorii* showed the greatest inhibition. To visualize the distribution of compounds and identify them in the inhibition zone produced by *P. cichorii*, we employed MALDI imaging mass spectrometry (MALDI IMS) and MS/MS molecular networking. We identified viscosin and massetolides A, F, G and H in the inhibition zone. Furthermore, viscosin was isolated and evaluated *in vitro* against *A. fumigatus* and *B. dendrobatidis* showing MIC values of 62.50 µg/mL and 31.25 µg/mL, respectively. This is the first report of cyclic depsipeptides with antifungal activity isolated from frog cutaneous bacteria.

## Introduction

Chytridiomycosis is a lethal infectious disease that is affecting amphibians worldwide. It is caused by the pathogenic fungi *Batrachochytrium dendrobatidis*^1–3^ and *B. salamandrivorans*^4^. *Batrachochytrium dendrobatidis* has caused the decline of a wide range of amphibians in several regions of the world including Australia, Southern Europe, North America and the Neotropical region. In contrast, *B. salamandrivorans* is thought to have originated in Asia, but has caused lethal outbreaks only in Europe and mainly affecting salamanders^4,5^. On the other hand, aspergillosis is a fungal disease that affect humans and other vertebrates^6–8^, most frequently caused by the opportunistic fungus *Aspergillus fumigatus*. In humans, this disease is generally treated successfully with traditional antifungal drugs. However, the rise of drug-resistant cases of aspergillosis^9^ is producing serious concerns in the medical community^10–12^, and the discovery of alternative antifungal drugs with different mechanisms of action is imperative.

Despite the amphibian declines caused by *B. dendrobatidis* in Central America and Panama^5^, some species are resistant to the fungal outbreaks and in some cases species may be recovering^13^. This resistance has been mainly attributed to chemical defenses comprised of antifungal skin secretions and secondary metabolites produced by epibiotic microbes on the skin of amphibians^14–19^. Although many antagonistic interactions between microbes and *B. dendrobatidis* have been described^15,20^, only four metabolites produced by amphibian cutaneous bacteria have been reported. They include violacein, indol-3-carboxaldehyde, 2,4-diacthylphloroglucinol, and prodigiosin^21–23^. Therefore, the chemical diversity of the compounds involved of these host and microbe interactions is still untapped, providing an opportunity for bioinspired antifungal drug discovery^9,10,12^. By exploring such novel natural sources of antifungals it is more likely to find different compounds with new mechanisms of action to enhance the therapeutic options for the control of fungal diseases^24^. In fact, during the last 30 years, only three main classes of molecules have been used in clinical practice including, polyenes, azoles, and echinocandins^25,26^. The latter are cyclic lipopeptides, which are molecules conformed by a fatty acid tail linked to a cyclic peptide. These molecules are of interest since they exhibit a broad spectrum of antimicrobial effects and have many biological roles in nature i.e., microbial antagonism, host-protection against predators, motility and biofilm formation^27,28^.

Modern technologies like DNA sequencing and mass spectrometry-based analyses, such as Matrix Assisted Laser Desorption Ionization (MALDI) Imaging Mass Spectrometry (IMS) and MS/MS molecular networking, can be used together to study microbial interactions and chemical ecology. Using high throughput sequencing of 16S rRNA gene amplicons, researchers can assess the array of bacterial species that are present in biological samples. While MALDI IMS facilitates direct visualization of the distribution of metabolites produced by interacting microbes in a Petri dish and MS/MS molecular networking is used to identify a wide range of known and unknown molecules with medical relevance^29–33^. By combining these techniques 1) 16S rRNA gene sequencing, 2) MALDI IMS and 3) Tandem Mass Spectrometry (MS/MS) molecular networking, it is possible to study the nature and dynamics of the production of chemicals in single microbes and during microbial interactions. Herein, we used this multi-omics approach to evaluate the potential of cutaneous bacteria from Panamanian frogs as a possible source of compounds to treat aspergillosis and chytridiomycosis.

## Results

### Sampling and bacterial isolation

Fifteen frogs of seven species (species identified in Table S1) were collected at three different sampling sites in the highlands of the Chiriquí Province, Republic of Panama (Fig. S1). These species were representatives of six anuran families: Craugastoridae (6), Centrolenidae (4), Hylidae (2), Bufonidae (1), Ranidae (1) and Strabomantidae (1). A total of 439 bacterial isolates were obtained from the skin of collected frogs, representing an average of 29.3 bacterial morphotypes per frog. Among sampling sites, specimens and isolates were obtained from Volcán, Fortuna Forest Reserve and La Amistad International Park with 8 frogs (250 isolates), 5 frogs (114 isolates) and 2 frogs (75 isolates), respectively (Table S1).

### DNA extraction and GTG_5_ rep-PCR fingerprinting

After cryopreservation, we obtained 432 viable bacterial isolates, of which DNA samples were extracted. From these samples, we identified 34 clusters by GTG_5_ rep-PCR with more than 91% band-matching similarity comprising 74% (319/432) of isolates. From these clusters, 136 isolates (4 per cluster) were selected for 16S rRNA analysis. Afterwards, we classified 25% (108/432) of samples that displayed fingerprint profiles between 70 and 90% band-matching similarities. From this last group of samples, 65 isolates were chosen for further 16S rRNA amplicon sequencing analysis (Fig. S2). Finally, isolates whose fingerprint values were lower than 70%, i.e., 1% (5/432), were excluded of taxonomic identification.

### 16S rRNA amplicon sequencing

**Figure 1:**
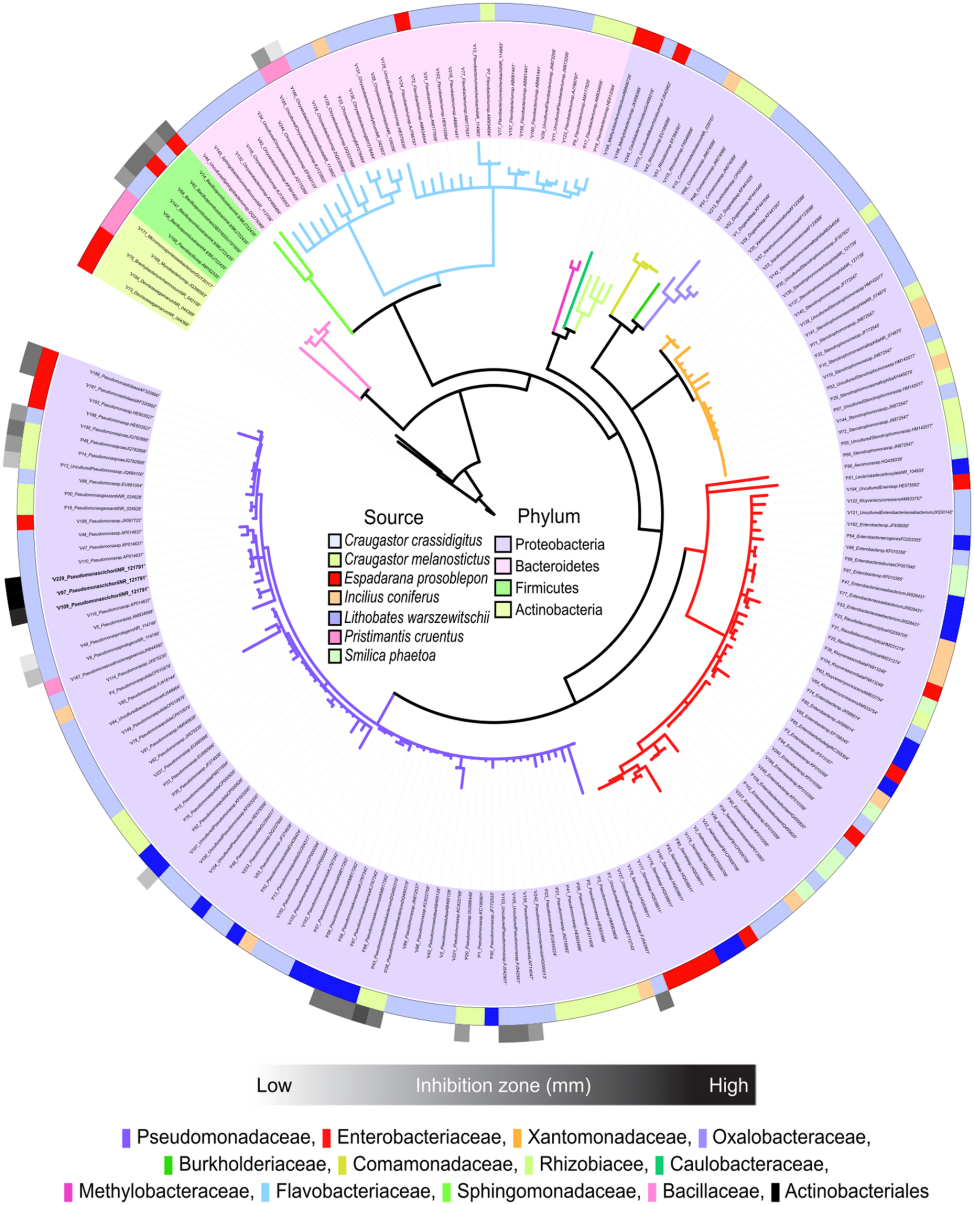
Phylogenetic tree based on 16S rRNA sequences of associated-bacteria from the skin of Panamanian frogs. Color range represents bacterial phyla. Color clades show the abundance of bacterial families. The color strips relate the bacterial isolates with their host of origin. Grey color scale strips display the inhibition zones produced by bioactive bacteria against *A. fumigatus* ATCC 1028 after microbial interactions.

Based on the fingerprints obtained from GTG_5_ rep-PCR analysis, 201 sequences from these strains were analyzed (Fig. S2). Strains belonged to the phyla Proteobacteria, Bacteroidetes, Firmicutes and Actinobacteria with a relative abundance of 78% (164), 17% (36), 3% (6) and 2% (4), respectively. Within the phylum Proteobacteria, the most abundant families were Pseudomonadaceae (39%), Enterobacteriaceae (21%), Flavobacteriaceae (16%) and Xanthomonadaceae (10%). However, the phyla Bacteroidetes and Firmicutes included the less abundant families Comamonadaceae, Oxalobacteraceae and Bacillaceae (2%), Methylobacteriaceae (1%) and Rhizobiaceae (1%). Families of the phylum Actinobacteria had a relative abundance of less than 1% (Fig. 1).

### Biological screening against *A. fumigatus* ATCC 1028

We found 29 of 201 isolates that displayed considerable biological activity against *A. fumigatus*. Active isolates included *Pseudomonas* spp. (22), *Bacillus* spp. (5) and *Chryseobacterium* spp. (2). The proportion of bioactive isolates per bacterial families comprises Pseudomonadaceae: 29.33% (22/75), Bacillaceae: 89.33% (5/6) and Flavobacteriaceae: 6.25% (2/32). In terms of hosts, 44.83% (13/29) of bioactive bacteria came from the skin of *Craugastor crassidigitus* (Craugastoridae), 20.69% (6/29) from the skin of *Craugastor melanostictus* (Craugastoridae), 13.79% (4/29) from each of the skin samples of *Espadarana prosoblepon* (Centrolenidae) and *Lithobates warszewitschii* (Ranidae), and 6.90% (2/29) from the skin of *Pristimantis cruentus* (Craugastoridae). Five out of 7 anuran species had bacteria with activity against *A. fumigatus*. 73% (21/29) of bacterial isolates that displayed this activity were obtained from frogs of family Craugastoridae. However, we did not find differences in bacterial isolates with activity among host families (Fisher’s Exact Test p = 0.567). The highest values of inhibition were shown by *Pseudomonas cichorii* (Blast identity score 99.90%), displaying inhibition zones between 13.33 ± 0.58 and 18.00 ± 0.00 mm. Such inhibition was two-fold more than the positive control cycloheximide (CHX) 9.00 ± 0.00 mm. In contrast, *Pseudomonas protegens* (Blast identity score 99.80%), had lower inhibition zones with values between 9.87 ± 0.58 and 10.67 ± 0.58 mm (Figs 1 and S3). Both bacterial species, *P. cichorii* and *P. protegens* were isolated from the skin of *C. crassidigitus*.

### Agar-based imaging mass spectrometry (MALDI IMS)

**Figure 2:**
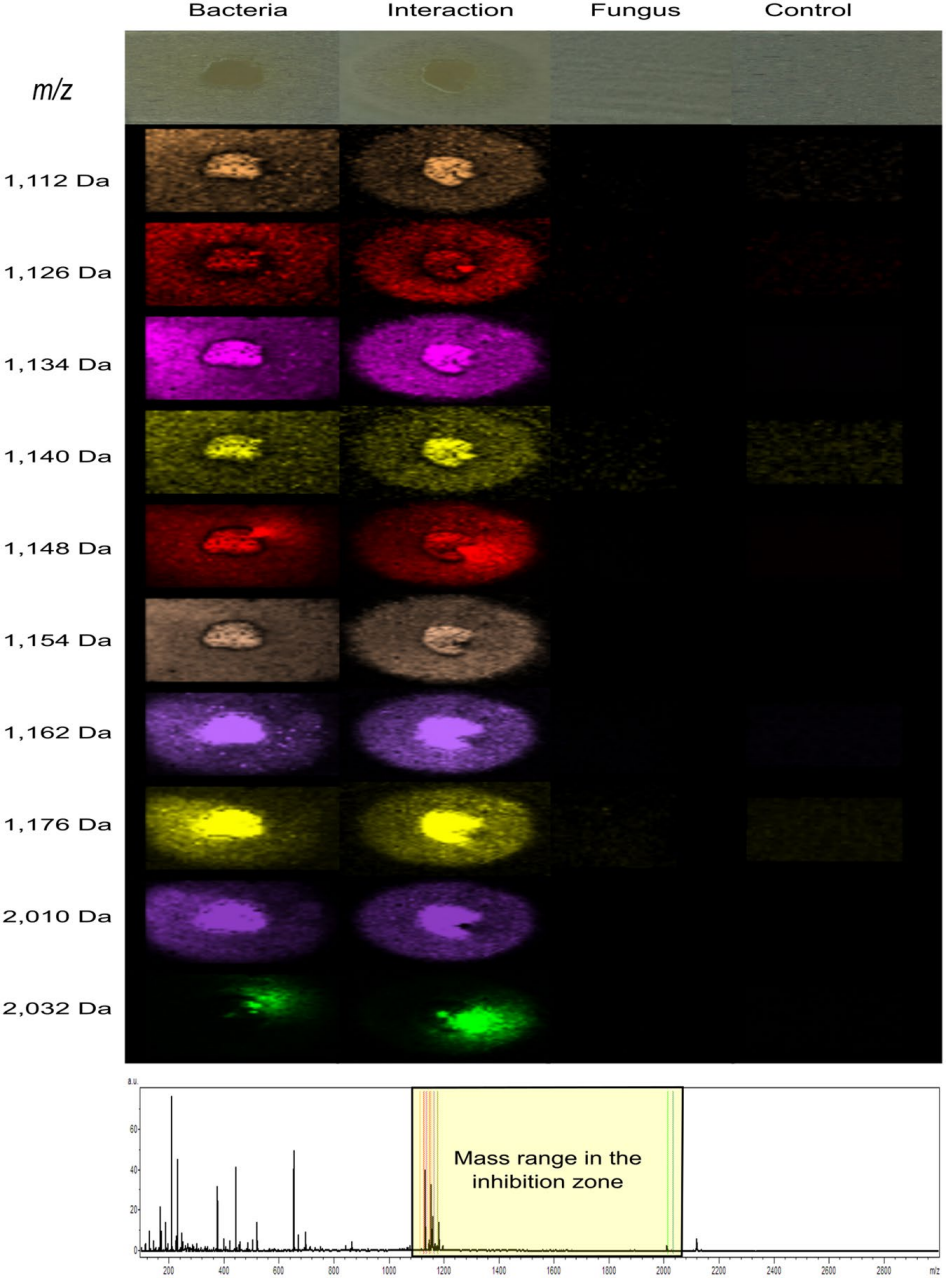
MALDI IMS of microbial interaction between *P. cichorii* and *A. fumigatus* ATCC 1028. Selected ions observed for the microbial interaction are presented as columns. The first column shows mass to charge ratio, and the second to fifth columns display images of the spatial distribution of the ions produced by *P. cichorii*, *P. cichorii* vs. *A. fumigatus*, *A. fumigatus* alone, and MHA as control. The average mass spectrum highlighting selected ions is shown at the bottom. These ions were present at m/z: 1,112, 1,126, 1,134, 1,140, 1,148, 1,154, 1,162, 1,176, 2,010 and 2,032 Da.

During the microbial interactions between *P. cichorii* and *A. fumigatus* ATCC 1028 we detected ten compounds specifically distributed in the inhibition zone using agar based MALDI IMS. They were detected as protonated [M + H]^+^ and sodium adduct [M + Na]^+^ molecular ions in the range *m*/*z* 1,110–2,032. These ions are 1,112 [M + H]^+^, 1,126 [M + H]^+^, 1,140 [M + H]^+^, 1,154 [M + H]^+^ and 2,010 [M + H]^+^ which matched their sodium adducts with *m*/*z* 1,134 [M + Na]^+^, 1,148 [M + Na]^+^, 1,162 [M + Na]^+^, 1,176 [M + Na]^+^, and 2,032 [M + Na]^+^, respectively (Fig. 2).

### MS/MS-based molecular networking

In MS/MS networking analyses, the molecules are subjected to MS^2^ fragmentation and are visualized as circles named nodes. The nodes are connected by edges, and the thickness of an edge indicates the similarity between the MS/MS spectra. A cluster is formed by nodes with similar MS/MS spectra, therefore they are structurally related. Identification of one compound in the cluster reveals the molecular family of the compounds.

**Figure 3:**
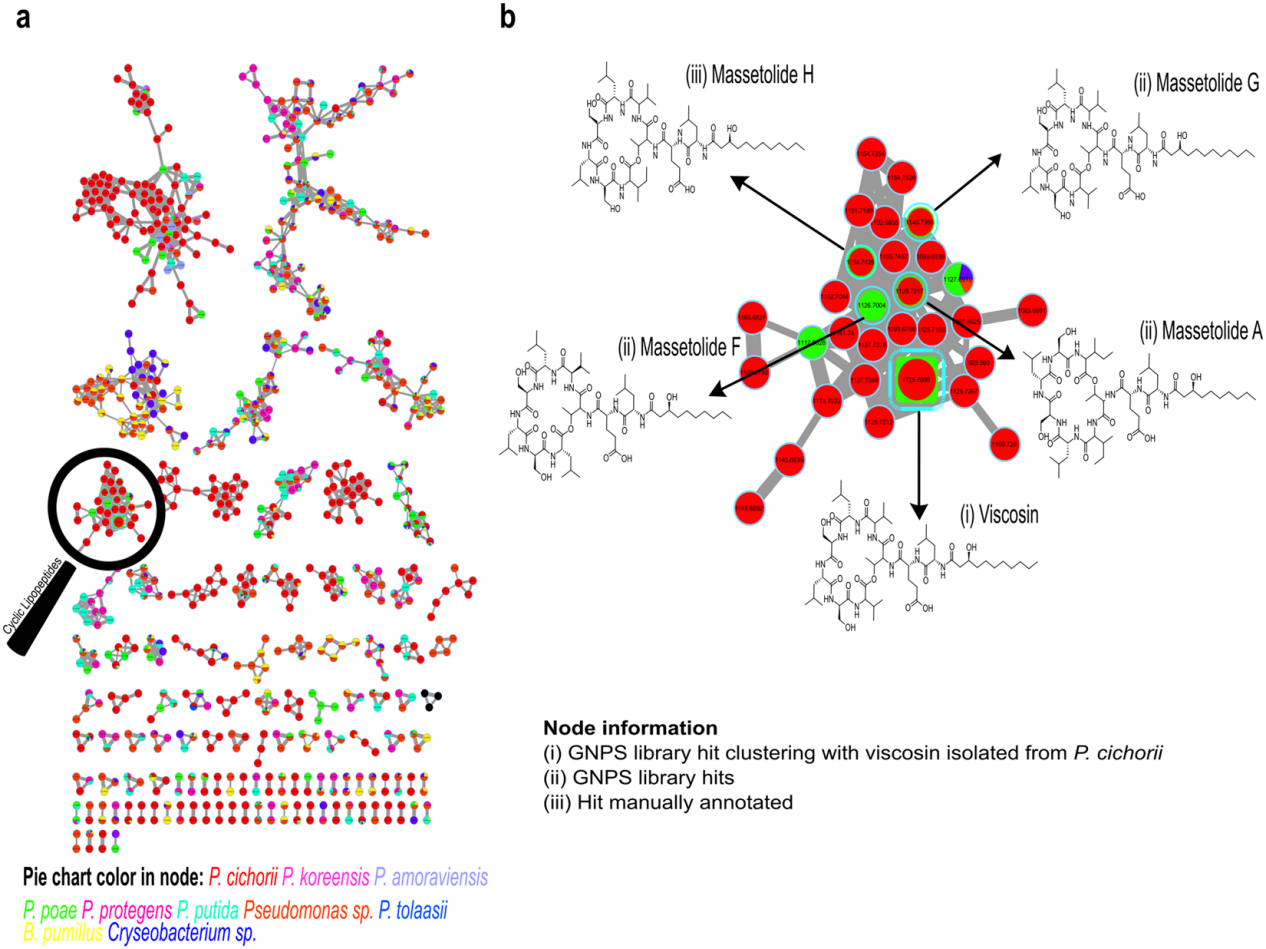
Molecular networking of bacterial strains that displayed biological activity against *A. fumigatus* ATCC 1028 after 72 hours. (**a**) Molecular networking of all bioactive strains against *A. fumigatus* ATCC 1028. Pie charts inside nodes represent the compound distribution based on the bacterial source. (**b**) Sub-network of cyclic lipopeptides detected inside inhibition zone through MALDI IMS. These nodes matched in GNPS spectral library. Square node comprises GNPS library hit WLIP/viscosin/massetolides molecular family and isolated viscosin from *P. cichorii*. massetolides A, F, G and H.

**Figure 4:**
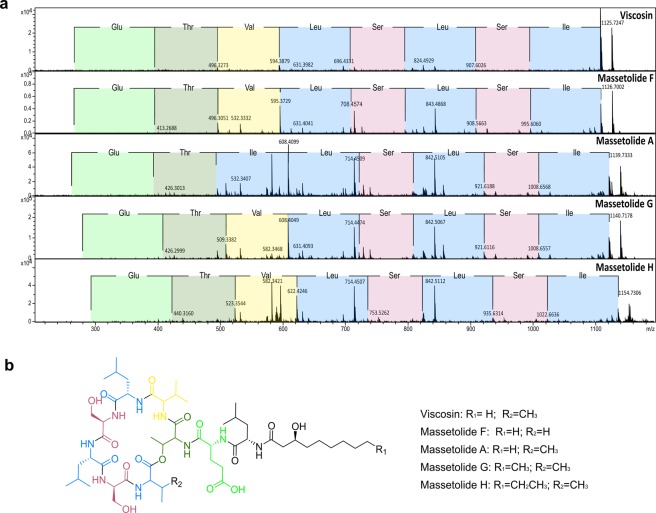
Spectral comparison and structural fragmentation of selected lipopeptides produced by bioactive bacteria. (**a**) MS/MS spectra of amino acid losses for viscosin, massetolide F, A, G and H. The color of the squares represents the amino acids in the spectrum. (**b**) Colored structural fragmentation of viscosin, massetolides F, A, G, H and their corresponding functional groups.

MS/MS molecular networking of the 29 bacterial strains with biological activity against *A. fumigatus* revealed 749 nodes. Among them, we focused on a specific cluster comprised of 30 nodes displaying four protonated molecules detected within the inhibition zone in the MALDI IMS experiment. These molecules were annotated as viscosin at *m*/*z* 1,125.71, massetolide F at *m*/*z* 1,126.68, massetolide A/G at *m*/*z* 1,140.74 and massetolide H at *m*/*z* 1,154. However, massetolide E at *m*/*z* 1,112.68 was not detected in samples from *P. cichorii*. It was detected in *P. poae* samples. These results indicated that this particular cluster is composed of multiple cyclic lipopeptides (Fig. 3). Annotation from Global Natural Products Social Molecular Networking online platform (GNPS)^34,35^ spectral library match and from the dereplicator tools was confirmed by detailed inspection of the fragmentation patterns observed in MS^2^ spectra (Fig. 4, Table S3).

### Extraction, purification and characterization of viscosin

After growing *P. cichorii* on Muller Hinton Agar (MHA), organic extraction with ethyl acetate (EtOAc) enabled us to obtain 22.0 mg of crude extract. This crude material was subsequently fractionated with a gradient of methanol (MeOH) and water (see methods). Viscosin (6.0 mg) was obtained and purified from the fraction at 100% MeOH using high performance liquid chromatography (HPLC) separation. It was identified by NMR experiments (Table S2)^28,36–38^. The MS/MS spectrum of viscosin displayed *m*/*z* 1,125.6968 [M + H]^+^ and the molecular formula for this compound was calculated for C_54_H_96_N_9_O_16_ with *m*/*z* 1,126.6970.

### Viscosin growth inhibition assays

**Figure 5:**
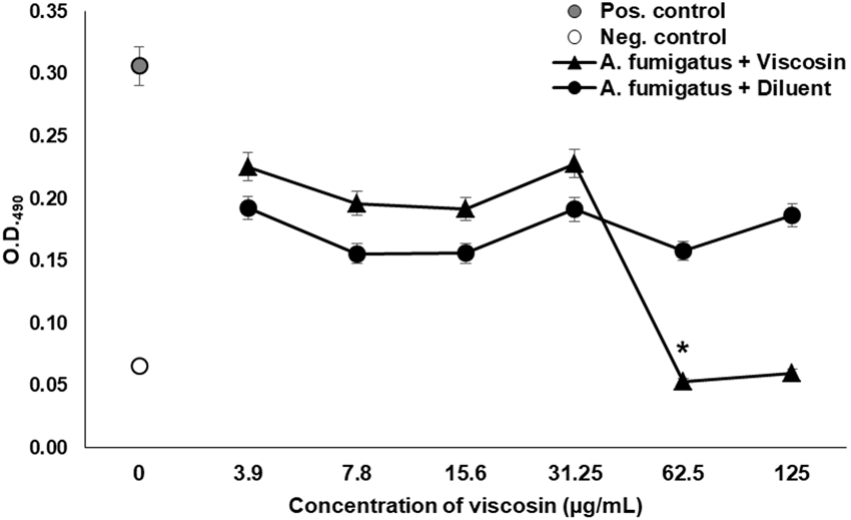
Antifungal susceptibility tests of viscosin against *A. fumigatus* ATCC 1028. Inhibition of growth of *A. fumigatus* by increasing concentrations of viscosin at 48 hours of culture. Viscosin displayed significant inhibition (*) when comparing with the positive control (p ≤ 0.0068) and diluent control at each concentration (p ≤ 0.0001) by Student’s t-tests. Error bars refer to the standard error of the mean. This figure is representative of two similar experiments. In both experiments, the MIC value was 62.50 μg/mL (Figs S4 and S5).

**Figure 6:**
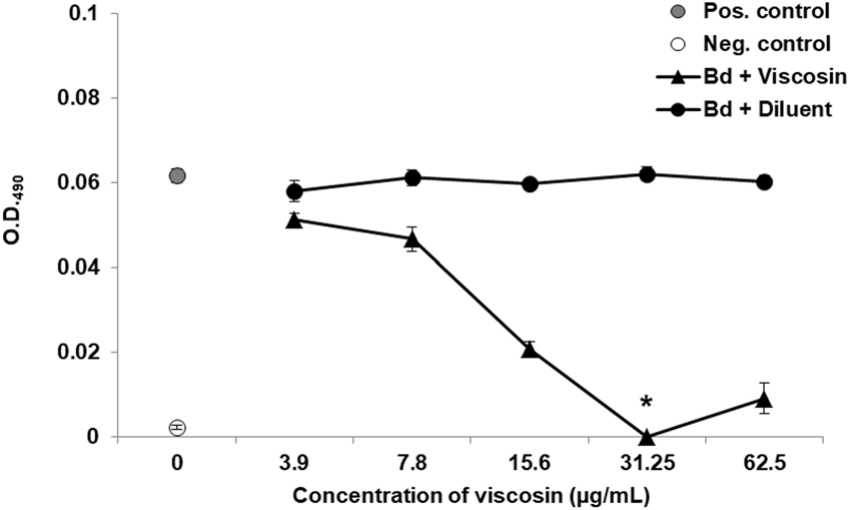
Antifungal susceptibility tests of viscosin against *B. dendrobatidis* JEL 197. Inhibition of growth of *B. dendrobatidis* zoospores by increasing concentrations of viscosin at day 4 of culture. Viscosin displayed significant inhibition (*) when compared with the positive control (p ≤ 0.0013) and diluent control at each concentration (p ≤ 0.05) by Student’s t-tests. Error bars refer to the standard error of the mean. This figure is representative of two similar experiments. In both experiments, the MIC was 31.25 μg/mL (Figs S6 and S7).

During the growth assay, we found that viscosin significantly inhibited *A. fumigatus* growth, compared to the positive control (t-test, p ≤ 0.0010) and the diluent control for every concentration tested (t-test, p ≤ 0.0130). Viscosin had a minimum inhibitory concentration (MIC) against *A. fumigatus* of 62.50 μg/mL (Fig. 5). In addition, we found significant inhibition of *B. dendrobatidis* growth by viscosin when compared with the positive control (t-test, p ≤ 0.0013) and when evaluated with the diluent control at each concentration (t-test, p ≤ 0.05) (Fig. 6). In this case, viscosin showed an MIC value of 31.25 μg/mL against *B. dendrobatidis*. The results shown are representative of two replicate experiments for each fungal species tested. In both cases, the MIC values were 62.50 μg/mL and 31.25 μg/mL for *A. fumigatus* and *B. dendrobatidis*, respectively (Figs S4–S7).

## Discussion

Chytridiomycosis is a lethal disease that affects amphibians worldwide, caused by the pathogenic fungus *B. dendrobatidis*^1–3,5^. Nonetheless, some amphibian species in affected areas have been shown to be able to persist in the presence of this fungus^13^. Although amphibians are known to produce antimicrobial peptides in the skin secretions produced by granular glands (also known as serous glands) in the dermal layer of the skin^39–44^, several studies have also suggested that amphibian species enduring the *B. dendrobatidis* outbreaks might have cutaneous bacteria that produce antifungal compounds that inhibit the growth of *B. dendrobatidis*^14–19^.

Within our sampling area of the highlands of the Chiriquí Province, *B. dendrobatidis* has caused dramatic declines in amphibian populations^5,45,46^. We collected 15 specimens from seven species to search for cutaneous bacteria with antifungal properties as a novel source of natural products. Our analysis showed that the phylum Proteobacteria is the most predominant phylum on the skin of sampled frogs as has been reported in other anuran species collected in Panama by using cultured organisms and culture-independent approaches^17,19,47,48^. Within the phylum Proteobacteria, Pseudomonadaceae was the more abundant family; nevertheless, among the less abundant phyla (Firmicutes, Bacteroidetes and Actinobacteria) there are remarkable differences in terms of family diversity, compared to previous studies^17,47^. Such differences could be attributable to the geographic location of sampling sites (e.g., highlands vs. lowlands, infected vs. non-infected sites), and how biotic and abiotic factors (e.g., frog species, habitat features, temperature) influence the bacterial communities on the skin of frogs^49^.

The families Pseudomonadaceae and Bacillaceae had the highest number of active bacteria against *A. fumigatus*. In particular, *P. cichorii*, isolated from the skin of *C. crassidigitus*, showed the strongest inhibition of *A. fumigatus* by agar diffusion assays. Remarkably, this inhibition was two-fold higher than our control cycloheximide (CHX)^50^. Afterward, we identified the molecules involved in this inhibition as cyclic lipopeptides through MALDI IMS and MS/MS molecular networking^32,33,35^. Through MALDI IMS experiments, we found that cyclic lipopeptides were produced when the bacterium was cultured in both, single culture and in co-culture with the fungus. It indicates that these compounds are not secreted as consequence of the microbial interaction, more likely they could be produced with a prophylactic purpose^51^. We report for the first time the presence of cyclic lipopeptides produced by skin-associated bacteria of Panamanian frogs. Among the cyclic lipopeptides produced by *P. cichorii*, we identified viscosin and massetolides (A, F, G, and H). Annotation of viscosin and massetolides was confirmed by conducting a detailed analysis of their MS^2^ spectra and matching them with those reported in the literature (Table S2)^28,36,38,52^.

Due to the problem of emerging multidrug resistant microorganisms, there is an increasing need for new drugs from uncommon bacterial sources to treat human and animal diseases. Thus, the molecules involved in the biological activity against *A. fumigatus* could potentially be a novel alternative to well-known bioactive molecules such as daptomycin^53^ and echinocandins (caspofungin, micafungin and anidulafungin), which have been approved by both the Food and Drug Administration (FDA) and the European Medicines Agency (EMEA) for use in human therapies^54,55^. Some of these molecules (i.e. caspofungin) exhibit inhibition of the β-glucan and chitin synthesis of the cell wall of *A. fumigatus*^56,57^. Although, Geuden *et al*.^58^ reported no inhibition of *A. fumigatus* growth by viscosin at concentrations used in their test (i.e., lower than 32 μg/mL), we found that viscosin, at 62.50 μg/mL, inhibited the growth of *A. fumigatus* by 80%. Therefore, we suggest future studies on viscosin as a possible β-glucan and chitin biosynthesis inhibitor, considering that these are essential components of the cell wall of *Aspergillus* spp.^57^.

Viscosin was also tested *in vitro* against *B. dendrobatidis*, showing a MIC value of 31.25 μg/mL. Until now, there are only a few secondary metabolites chemically characterized that display inhibitory effects against *B. dendrobatidis*. These compounds are 2,4-diacetylphloroglucinol, indole-3-carboxaldehyde, violacein and prodiogiosin^21–23,59^. Although viscosin is known for its antibiotic effects^58,60^, it had not been previously isolated from cutaneous bacteria of amphibians nor tested for effects on *B. dendrobatidis*. Future studies on chitin synthesis inhibition by viscosin-like lipopeptides could be an interesting subject of research, taking into account that chitin is the major component of chytridiomycete cell walls^61,62^. In addition, although not tested in this study, there is the potential that multiple molecules act synergistically to fend off pathogens^63–67^. Isolation and testing of the *in vitro and in vivo* (using frogs inoculated with *B. dendrobatidis*) antifungal activity of massetolides, singly and in combination with viscosin, could be the next step of this study, since this compounds were found together at the inhibition zone of the microbial interaction, and their synergistic action might increase their biological activity.

The data shown here support the hypothesis on the protective role mediated by cultivable bacteria that include a viscosin-producing strain, on the skin of some anurans through the production of antifungal lipopeptides. This might help to explain the resistance of some amphibian species to *B. dendrobatidis* infection.

## Conclusions

We conclude that the family Pseudomonadaceae in the phylum Proteobacteria represents the most abundant cultivable bacterial taxa found on the skin of the frogs sampled for this study. We also found that Pseudomonadaceae and Bacillaceae were the sources of the major number of antifungal bioactive bacteria. Among these bacteria, *P. cichorii* displayed the highest inhibitory activity against *A. fumigatus*. By means of 16S rRNA sequencing, MALDI IMS and MS/MS molecular networking, it was possible to detect cyclic lipopeptides produced by *P. cichorii*. One of them was viscosin that displayed *in vitro* biological activity against both *A. fumigatus* and *B. dendrobatidis*, inhibiting their growth with MIC values 62.50 and 31.25 µg/mL, respectively. This work can be used as basis for future *in vivo* studies treating amphibians inoculated with *B. dendrobatidis*. This work provides evidence on cutaneous bacteria in Panamanian frogs as an underexplored source of compounds to treat fungal diseases such as aspergillosis and chytridiomycosis.

## Methods

### Sampling and bacterial isolation

Fifteen frogs of seven species were collected at three localities in the highlands of the Chiriquí Province, Republic of Panama (Fig. S1, Table S1). To remove transient bacteria from the skin of frogs, all specimens were rinsed with sterile water^47,68^. After rinsing, a sterile swab was used for obtaining culture-dependent bacteria. This swab was streaked on Petri dishes containing fresh R2A agar^69,70^ (Becton, Dickinson and Co., New Jersey, US). After sampling, the frogs were identified and released immediately at the collection place. Once bacterial colonies grew, they were characterized according to morphological features. Then, isolates were streaked on R2A agar until pure colonies were obtained. The pure isolates were cryopreserved at −80 °C in R2A broth supplemented with 15% glycerol. Sampling of bacteria used in this study was carried out under collection permit SC/AHB-1–11 granted by the Ministry of Environment of Panama (MiAMBIENTE).

### 16S rRNA amplicon sequencing

After DNA extraction and GTG_5_ rep-PCR fingerprinting analysis (see supplementary information), two universal primers 27 F (5′-AGAGTTTGATCCTGGCTCAG-3′) and 1492 R (5′- GGTTACCTTGTTACGACTT-3′) were used in PCR to amplify the 16S rRNA gene for the identification of 202 bacterial samples. The amplification reactions were carried out in 50 µL (45 µL of Master Mix and 5 µL of bacterial DNA). The reaction mixtures were amplified at 95 °C for 5 min, followed by 30 cycles of 94 °C for 1 min, 55 °C for 1 min, 72 °C for 90 sec, and a final elongation for 10 min. PCR products were checked by electrophoresis in Agarose gel 1%. Amplicons were sent to Macrogen Inc. (Seoul, South Korea) for Sanger sequencing. Furthermore, DNA sequences were cleaned, assembled and analyzed with Geneious 8.1.7 (Biomatters, Auckland, New Zealand)^71^. Additionally, a phylogenetic tree was created with Neighbor-Joining as clustering method (bootstrap of 1000 replicates). It was exported from Geneious (Newick format) and imported to the online platform ITOL for data visualization^72,73^. The final tree is available at (https://itol.embl.de/tree/20046278967671452714596#). The 16S taxonomic diversity was obtained from the Ribosomal Database Project (RDP) and BLASTn. Sequences are available in GenBank with accession codes KX456226-KX456434.

### Agar-based imaging mass spectrometry (MALDI IMS)

MALDI IMS was conducted after screening all bacteria against *A. fumigatus* (see supplementary information). The most bioactive bacteria were analyzed by using a thin layer of Mueller Hinton agar (MHA). Individually, Petri dishes had: 1) bacterial colony of *P. cichorii*, 2) microbial interaction of *P. cichorii* vs. *A. fumigatus*, 3) *A. fumigatus* ATCC 1028 and 4) MHA as control. The agar in Petri dishes was cut and placed onto a ground steel MALDI MTP plate (Bruker Daltonics, Billerica, MA, USA). This MALDI plate was covered with the universal MALDI matrix (1 α-Cyano-4-hydroxycinnamic acid HCCA 1:1 mixture, Sigma Aldrich Catalog 50149) by a dry deposition method using a molecular sieve of 44 μm and dried overnight at 37 °C. A photograph was taken before and after adding/drying universal MALDI^74^. MALDI IMS was conducted using an UltrafleXtreme mass spectrometer (Bruker, Bremen, Germany) for mass spectra acquisition. Samples were run in positive reflector mode at 500 μm laser intervals in XY and a mass range of 100–3000 Da. Results were analyzed by FlexImaging 3.0 software (Bruker). Images of interesting ions were color-generated, exported and organized using ImageJ 1.47 V software (NIH, Bethesda, MD).

### MS/MS-based molecular networks

Organic extracts from bioactive bacteria were analyzed through liquid chromatography-tandem mass spectrometry (LC-MS/MS) experiments by electrospray ionization quadrupole-time of flight mass spectrometry (ESI-Q-TOF-MS) in a micrOTOF-QIII mass spectrometer (Bruker). The liquid chromatography method selected for analysis was conducted in a 1290 Infinity UHPLC equipment (Agilent Technologies, Santa Clara, CA), using a KINETEX reverse phase column with C-18 chemistry, 50 mm × 2.1 mm and 1.7 μm particle size (Phenomenex, Torrance, CA). The mobile phase was 0.1% formic acid in Milli-Q water in channel A and methanol in channel B. Liquid chromatography runs were performed with a gradient of 10 to 100% B in 7 min with additional isocratic 100% B for 9 min. Mass spectrometry data were acquired in positive ion mode with a detection range of 50–2250 m/z using data dependent MS/MS fragmentation^75^.

Molecular networks were generated using the Global Natural Products Social Molecular Networking online platform (GNPS)^34,35,76^. The MZmine workflow for feature based molecular networking on GNPS was used to perform feature detection, grouping, and alignment^77,78^ (see supplementary methods). MS-Clustering was turned off, and a spectral library search was performed using 0.02 Da for ion mass and fragment ion tolerance. A cosine score for getting spectral matching with MS/MS spectral libraries was set at 0.70. A minimum of four matching peaks was considered for spectral library annotations. The resulting molecular network is available at https://proteomics2.ucsd.edu/ProteoSAFe/status.jsp?task = cbd4d0d6e4b34a05a0e29ec542750d5a. This network was imported to Cytoscape version 3.5.0 (www.cytoscape.org) and analyzed using default algorithms and other visual considerations. An ion mass tolerance and a fragment ion mass tolerance of 0.02 Da were used. The number of minimum amino acids was set to five. The dereplication process was achieved by searching MS/MS spectra into GNPS libraries. This result was imported in the network generated in Cytoscape 3.5.0. The MS/MS spectra of annotated compounds are publicly accessible at GNPS library through the accession numbers: CCMSLIB00000579932, CCMSLIB00000579933, CCMSLIB00003742128 and CCMSLIB00000579934. Dataset is available in Massive (MSV000082747) at https://massive.ucsd.edu/ProteoSAFe/status.jsp?task = ef5e14c452ee403eb1d69cfae55a8895.

### Extraction, purification and characterization of viscosin

To identify ions of interest detected in MALDI IMS experiments, *P. cichorii* was grown in single culture using 30 square dishes (120 × 120 mm) continaing MHA for 72 hours at room temperature. Agar was cut in strips and placed into 2000 mL Erlenmeyer flasks for maceration with ethyl acetate (EtOAc) for 24 hours. Strips were extracted with EtOAc (3 × 500 mL) and resuspended in methanol (MeOH) for fractionation and MS analysis.

The extract obtained was fractionated using a Superclean LC 18 cartridge (Catalog 57136, Supelco Analytical, Sigma-Aldrich) eluted with 20%, 40%, 60%, 80% and 100% of MeOH in H_2_O^79^. Afterwards, each fraction and crude extract was analyzed in LC-MS/MS for detection of the presence of lipopeptides.

Compound separation was carried out on Agilent 1100 HPLC. Fraction E (100% MeOH) was injected in a Synergi 4 u Polar C18 semi-preparative column (250 × 10 mm) of 4 µm particle size (Phenomenex, Torrance, CA) applying a gradient elution method from 80:100% MeOH/H_2_O in 30 min, and operating at 1 mL/min with a diode array detector (DAD) set for single-wavelength detection at 254 nm. The peak of interest was re-injected into the same column with a gradient elution from 50:50 to 100% acetonitrile/water in 20 min with 0.1% trifluoroacetic acid (TFA), and operating at 4.2 mL/min with a DAD set for single-wavelength detection at 214 nm^38^. NMR spectra of the sample were obtained in a Jeol Eclipse 400 MHz spectrometer and referenced to residual solvent ^1^H and ^13^C signals (δ_H_ 3.31, 4.87 and δ_C_ 49.15 for C).

### Viscosin growth inhibition assays

Viscosin was evaluated against *A. fumigatus* in a range of 0.4 to 5 × 10^4^ CFU/mL, according to the reference method for broth dilution antifungal susceptibility testing of filamentous fungi^80^. The final concentration of viscosin in the wells ranged from 3.90 to 500 µg/mL. The trays were incubated at 35 °C, and read after 48 hours at 490 nm (O.D._490_)^81^.

In the case of *B. dendrobatidis*, mixed zoosporangia and zoospores of isolate JEL197 were plated onto 1% tryptone agar plates, and cultured at 21 °C for 3 days. Zoospores were purified by twice flooding the agar with sterile 1% tryptone broth^82^. The broth containing zoospores was filtered with a 20 µm mesh opening filter, and isolated zoospores were resuspended in 1% tryptone broth at a concentration of 1 × 10^6^/mL. Freshly isolated zoospores were plated in five replicate wells (5 × 10^4^/50 µL) in 1% tryptone broth with 50 µl of a serially diluted viscosin at concentrations in culture ranging from 3.9 µg/mL to 250 µg/mL. The plates were incubated at 21 °C for four or seven days, and growth was measured as an increase in optical density at 490 nm (O.D._490_)^83^.

For these assays, viscosin (1.1 mg) was dissolved in 100 µL of 70% ethanol. The viscosin was resuspended in a total volume of 1.1 mL with sterile water plus penicillin (100 I.U/mL) and streptomycin (100 µg/mL) to equal a stock suspension of 1 mg/mL. The stock was further diluted serially with sterile water and antibiotics to 500, 250, 125, 62.5, 31.25, 15.6, 7.8 and 3.9 µg/mL, and 50 µL of each dilution (five replicates) was added to 50 µL of target fungi. To determine the possible effects of the ethanol diluent, serial dilutions were prepared without viscosin, and fungi were cultured independently with each dilution of ethanol. Ethanol concentrations ranged from 0.03 to 1.75%. Positive control wells (without viscosin or ethanol) contained fungi and 50 µL of sterile water plus antibiotics. Negative control wells contained 50 µL of heat-killed fungi (10 min at 60 °C for *B. dendrobatidis* and 100 °C for *A. fumigatus*) and 50 µL of sterile water plus antibiotics.

## Supplementary information

Viscosin-like lipopeptides from frog skin bacteria inhibit Aspergillus fumigatus and Batrachochytrium dendrobatidis detected by imaging mass spectrometry and molecular networking

## Data Availability

All data generated or analyzed during this study are included in this article and in it supporting information files. Acquired MS data is deposited in the GNPS MassIVE repository under the accession number: MSV000082747 (see link in methods). Additionally, all sequences are deposited in Genbank under the accession codes KX456226-KX456434.
