# Supplementary material for: Viscosin-like lipopeptides from frog skin bacteria inhibit Aspergillus fumigatus and Batrachochytrium dendrobatidis detected by imaging mass spectrometry and molecular networking
Source: Sci Rep. 2019 Feb 28;9:3019. doi: 10.1038/s41598-019-39583-7 (PMC6395710; doi:10.1038/s41598-019-39583-7)
Supplement: Supplementary file 1 — Viscosin-like lipopeptides from frog skin bacteria inhibit Aspergillus fumigatus and Batrachochytrium dendrobatidis detected by imaging mass spectrometry and molecular networking [file 41598_2019_39583_MOESM1_ESM.pdf]

Viscosin-like lipopeptides from frog skin bacteria inhibit *Aspergillus fumigatus* and *Batrachochytrium dendrobatidis* detected by imaging mass spectrometry and molecular networking

Christian Martin H [1,2], Roberto Ibáñez [3,4], Louis-Felix Nothias [5], Cristopher A. Boya [1,2], Laura K. Reinert [6], Louise A. Rollins-Smith [6,7], Pieter C. Dorrestein [5] and Marcelino Gutiérrez [1*]

[1]Center for Biodiversity and Drug Discovery. Institute for Scientific Research and Technology Services (INDICASAT AIP), Clayton, Panama, Republic of Panama

[2]Department of Biotechnology, Acharya Nagarjuna University, Guntur, India

[3]Smithsonian Tropical Research Institute, Balboa, Ancon, Republic of Panama

[4]Departamento de Zoología, Universidad de Panamá, Panama, Republic of Panama

[5]Collaborative Mass Spectrometry Innovation Center, Skaggs School of Pharmacy and Pharmaceutical Sciences, University of California San Diego, La Jolla, California, USA

[6]Department of Pathology, Microbiology, and Immunology, and Department of Pediatrics, Vanderbilt University School of Medicine, Nashville, Tennessee, USA

[7]Department of Biological Sciences, Vanderbilt University, Nashville, Tennessee, USA

[*]Correspondence:

Marcelino Gutiérrez, Center for Biodiversity and Drug Discovery; INDICASAT AIP. Building 219, City of Knowledge, Clayton, Panama, Republic of Panama.

PO Box 0843-01103

mgutierrez@indicasat.org.pa
Phone: (507) 5170700
Fax: (507) 5070020

Supplementary information.

Contents

- **Supplementary methods**
- **Supplementary figures**
- **Supplementary tables**
- **Supplementary references**

Supplementary methods

DNA extraction and GTG_5 rep-PCR fingerprinting

Rep-PCR fingerprinting was used in this study for rapid grouping and tentative identification of microbes to avoid multiple 16S RNA sequencing of the same strains. Bacterial DNA contains repetitive elements distributed over their genome and primers used through this technique could anneal such elements. PCR products are separated by agarose gel electrophoresis and specific band-patterns or fingerprints are obtained. Similar fingerprints will cluster together and full identification of a limited number of isolated could be reach by 16S rRNA gene sequencing.

Aliquots of 500 µL 5% Chelex®100 resin were placed in 1.5 mL microcentrifuge tubes (Eppendorf, Hamburg, Germany). A cluster of rapidly growing bacterial cells were then introduced and gently vortexed. All tubes were incubated at 56 °C for 20 min, boiled for 10 min, and placed in crushed ice for 2 min. Tubes were then vortexed for 10 seconds and centrifuged at 13,000 rpm for 5 min. Supernatants containing DNA were carefully passed into new 1.5 mL tubes. DNA concentrations were measured using a

Nanodrop 2000/2000c spectrophotometer (Thermo Fisher Scientific, Waltham, MA) for Polymerase Chain Reaction (PCR) experiments [1].

DNA samples (432) were subjected to rep-PCR genomic fingerprinting using a single oligonucleotide primer GTG_5 (5′-GTGGTGGTGGTGGTG-3′). The reactions were performed in 25 µL (22 µL of Master Mix and 3 µL of bacterial DNA). PCR was conducted in a thermocycler T3000 (Biometra, Gottingen, Germany) at 94 °C for 5 min (activation), followed by 35 cycles of 94 °C for 45 seconds (denature), 40 °C for 1 min (anneal) and 65 °C for a period of 10 min (extension), and a final elongation for 20 min at 65 °C. All PCR products were electrophoresed in 1.2% Agarose gel (Catalog A6877, Sigma-Aldrich, St. Louis, MO) in a 1X Tris/Borate/EDTA solution (TBE) for 3.5 hours at 145 volts. The rep-PCR profiles were visualized under ultraviolet light after staining with ethidium bromide (EtBr 0.5 µg/mL). Digital images were captured using an Ultra-Lum camera cabinet (Artisan Technology Group, Champaign IL, USA) and analyzed through BioNumerics software V6.6 (Applied Maths, Ghent, Belgium). This analysis was based on Pearson correlation and UPGMA as clustering method. Data was exported in Newick format and imported into the online platform Interactive Tree Of Life (http://itol.embl.de) for visualizing the resulting tree (Figure S2) [2,3].

Biological screening against *A. fumigatus* ATCC 1028

Aspergillus fumigatus ATCC 1028 was grown for 7 days on Potato Dextrose Agar (PDA) at 30 °C. Then, a conidia suspension in saline solution was prepared at 0.80-1.00 x 10^5 CFU/mL. Subsequently, it was streaked on Petri dishes with Muller Hinton Agar (MHA). In parallel, selected isolates (Figure S2) were grown for 24 hours at 0.50 McFarland (1.00 x 10^6 CFU/mL). Afterwards, bacterial cultures of each sample were

placed on Petri dishes in co-culture with *A. fumigatus* ATCC 1028 and incubated at room temperature for 72 hours. Cycloheximide (CHX) is commonly used as fungicide in *in vitro* research [4]. Therefore, CHX at 10 μg/mL was used as negative control for growth and ethanol 95% (EtOH) as a positive control. Bacterial cultures that displayed clear inhibition zones were considered as bioactive. Three replicates of each bioactive isolate was analyzed. The isolate with the strongest bioactivity (18.00 mm) was analyzed by MALDI IMS.

MZmine workflow on GNPS

Molecular networking through Global Natural Products Social Molecular Networking (GNPS; http://gnps. ucsd.edu), is a tandem mass spectrometry (MS/MS) data organizational approach that have been recently introduced in different fields such as drug discovery, metabolomics, clinical and forensic toxicology [5,6]. Molecular networks are visual displays of the chemical space present in tandem mass spectrometry (MS/MS) experiments. Thus, researchers can detect sets of spectra from related molecules. Molecular networks in GNPS represents each spectrum as a node, and spectrum-to-spectrum alignments as edges (connections) between nodes [7].

All mass spectrometry data files must be in a compatible, universal format (mzXML, mzML or mgf). For this study, mass spectrometry data as mzXML files were processed with MZmine workflow on GNPS [7]. The MZmine parameter of the workflow for LC-MS was as follows: Feature extraction for MS^1 was achieved with a signal threshold of 2.0 $x10^3$, and 1.0 $x10^3$ for MS^2. Chromatogram builder was run with a minimum height of 1.5 $x10^3$ and tolerance of 25 ppm. Chromatograms were deconvoluted with a peak duration range of 0.025 to 2.0 min and a baseline cut-off

algorithm of 2.0 x10^3. Detected peaks were aligned through Join Aligner Module considering mass (15 ppm) and retention time tolerance (0.35 min).

Molecular network are visualized online in-browser or exported for visualization to Cytoscape software. Once molecular network is exported, Cytoscape applies defaults clustering algorithms as layouts (force-directed layout). It consists on repulsive forces between all graph nodes and attractive forces between adjacent nodes. Cosine scores define attractive forces between nodes and determines the similarity of two MS/MS spectra with scores ranging from 0 (totally dissimilar) to 1 (completely identical).

Figure S1. Sampling sites at three different locations in the province of Chiriquí in the Republic of Panama. a) Fortuna Forest Reserve , b) Volcán near to the Pozos Termales , and c). La Amistad International Park (Las Nubes Ranger Station).

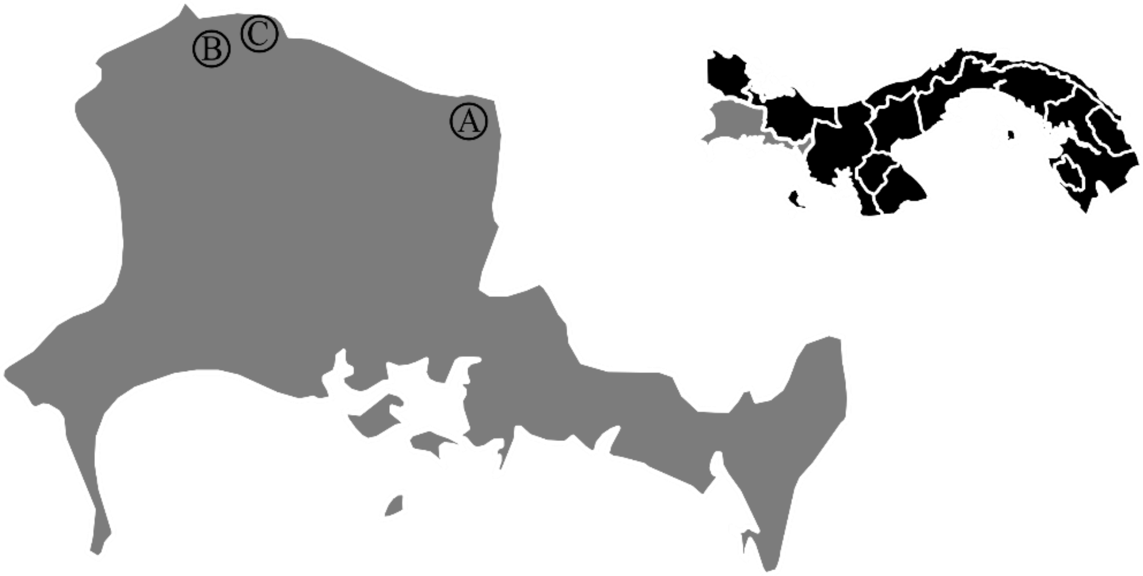

Figure S2. Comparison dendogram of GTG_5 rep-PCR for 432 isolates obtained from the skin of frogs collected at three sampling sites in Chiriquí Province, Panama. Fortuna Forest Reserve (blue), Volcán near to the Pozos Termales (yellow) and La Amistad International Park (Las Nubes Ranger Station) (red). Collapsed clades represent 34 clusters with more than 91% band-matching similarity. Samples from each clade (N=4) and those marked with black squares (70-90% fingerprint similarities) were chosen for 16S rRNA amplicon sequencing. Sample marked with red square was removed from selection after presenting low quality in their DNA sequence.

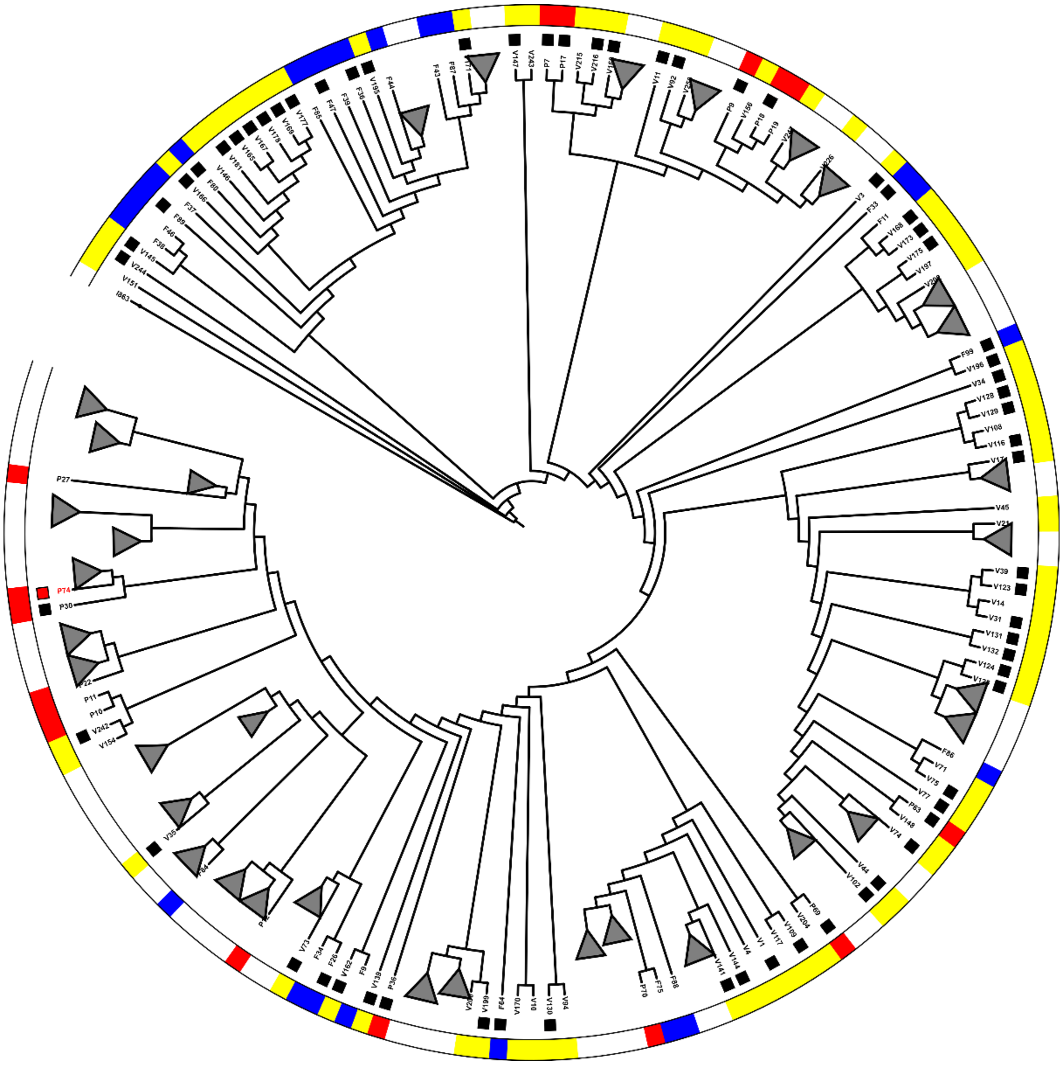

Figure S3. Bar chart of agar diffusion assay (n=3 replicates) representing the inhibition zones (mm) displayed by skin-associated isolates of collected frogs against *A. fumigatus* ATCC 1028. Negative control for growth was CHX (10 μg/mL and the positive control for growth was ethanol 95%.

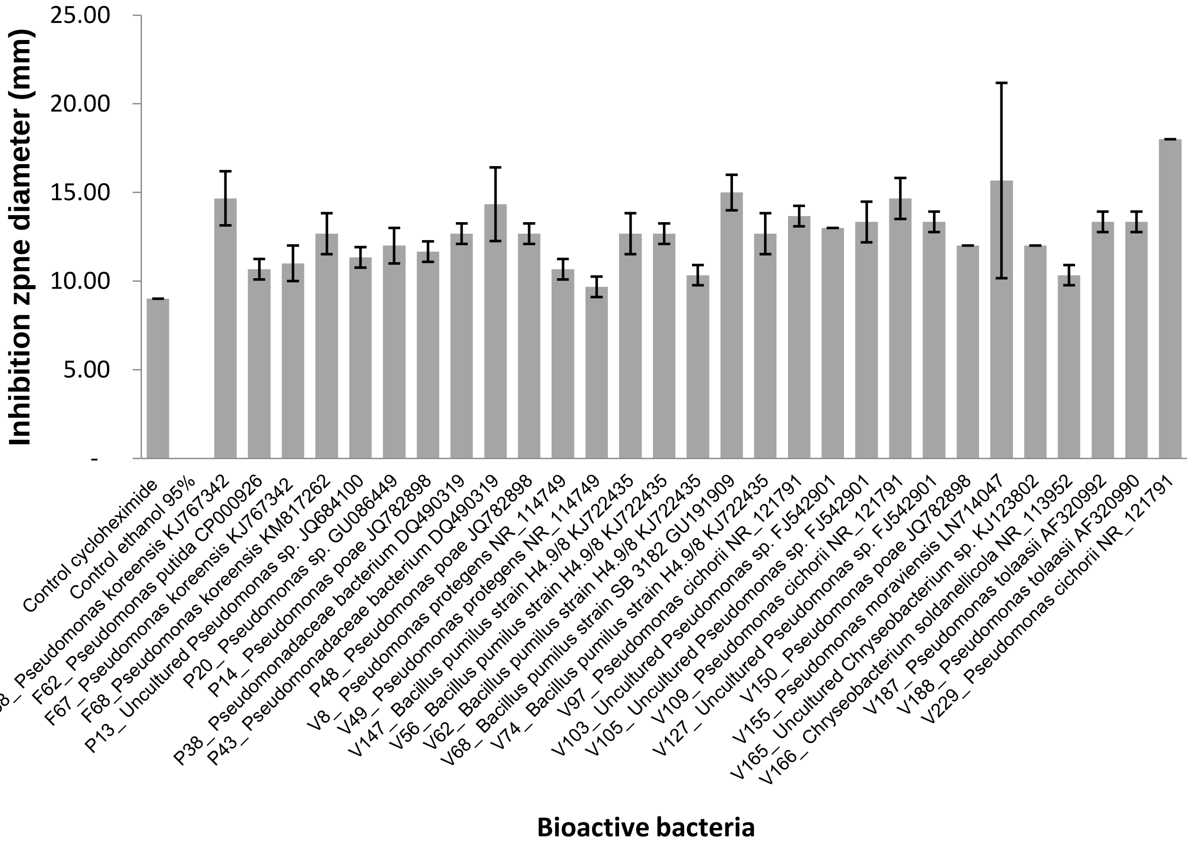

Figure S4. Inhibition of growth of *A. fumigatus* by increasing concentrations of Viscosin at day 48 hours of culture. Experiment 1.

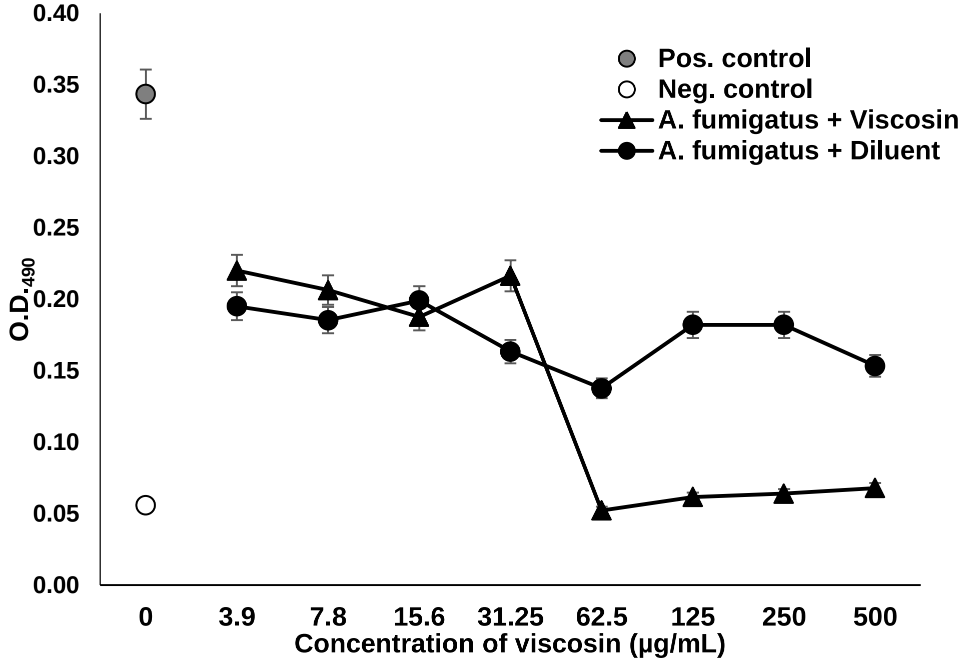

Figure S5. Inhibition of growth of *A. fumigatus* by increasing concentrations of Viscosin at day 48 hours of culture. Experiment 2.

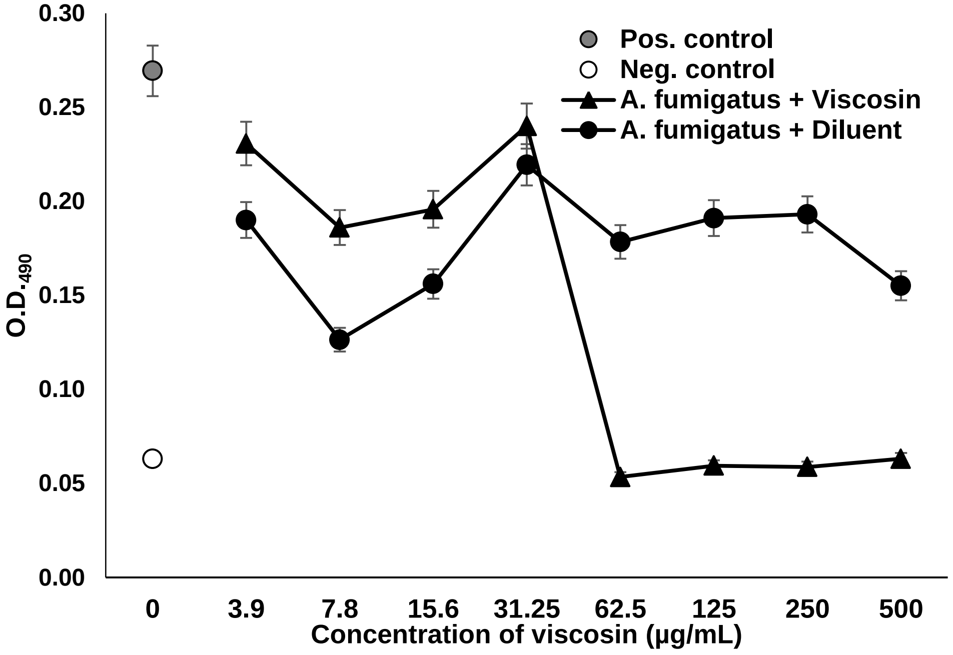

Figure S6. Inhibition of growth of *B. dendrobatidis* zoospores by increasing concentrations of Viscosin at days 4 and 7of culture. Experiment 1. In this experiment, we note that higher concentrations of viscosin increased O.D. above the MIC value. We attribute this to turbidity because viscosin is not completely soluble at these concentrations.

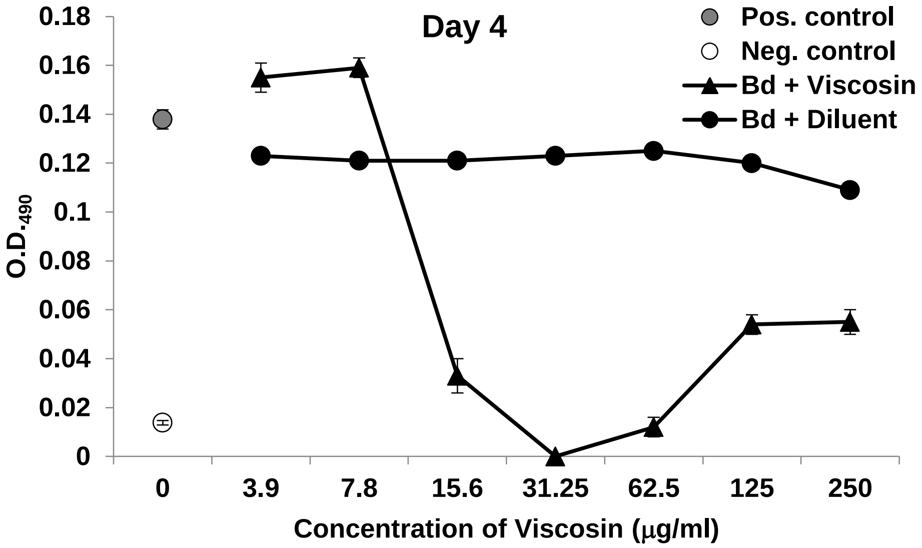

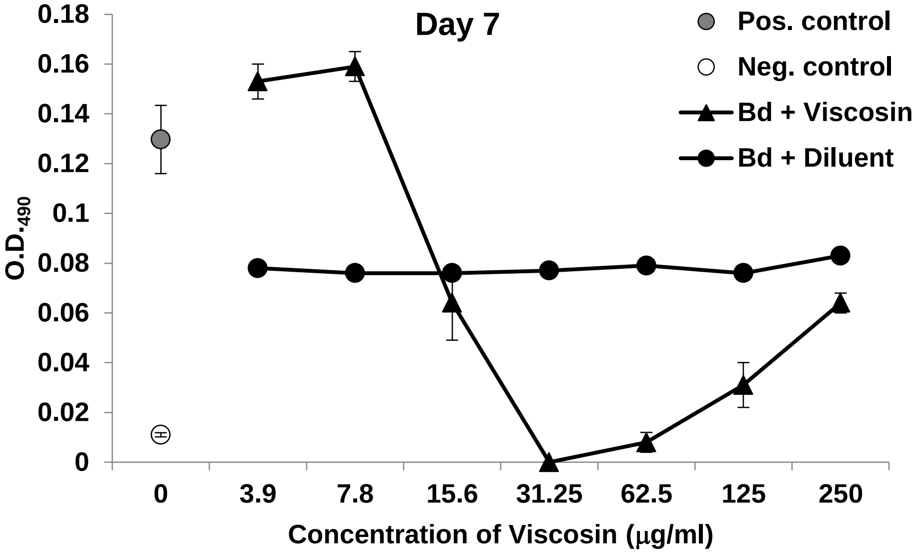

Figure S7. Inhibition of growth of *B. dendrobatidis* zoospores by increasing concentrations of Viscosin at days 4 and 7 of culture. Experiment 2. In this experiment, there was a possible contamination at day 7. However, zoospores were all dead at viscosin concentrations above 31.25 µg/ml.

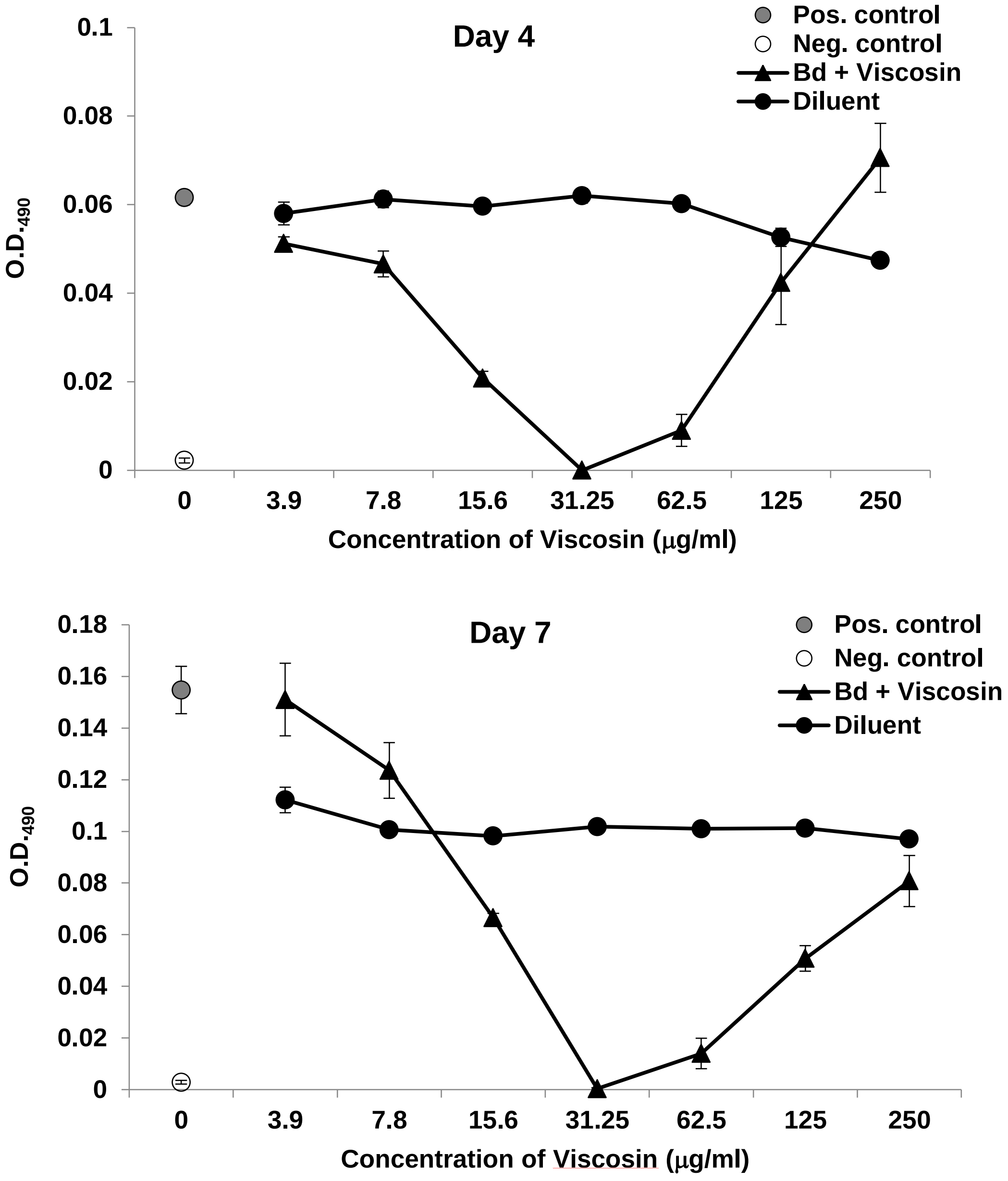

Supplementary tables

Table S1. Number of bacterial isolates from the skin of each species of Panamanian frogs collected at three sites in the highlands of Chiriquí.

Sampling site	Number of frogs	Frog species (number of individuals)	Number of isolates (range per frog)
**Fortuna Forest Reserve**	5	*Incilius coniferus* (1)	36
		Espadarana prosoblepon (1)	8
		*Lithobates warszewitschii** (1)	39
		Smilisca phaeota (2)	31 (5-26)
**Volcán**	8	*Craugastor crassidigitus* (4)	198 (11-64)
		Espadarana prosoblepon (3)	43 (9-19)
		Pristimantis cruentus (1)	9
**La Amistad International Park**	2	*Craugastor melanostictus* (2)	75
**Total**	**15**		**439**

* Infected by *Batrachochytrium dendrobatidis*.

Table S2. Comparison of NMR Data of the isolated Viscosin (**1**), and Viscosin data from literature (CD_3OD, TMS as internal reference).

	Compound 1; ^{1}H δ[ppm]	Viscosin; ^{1}H δ[ppm]	Compound 1; ^{13}C δ[ppm]	Viscosin; ^{13}C δ[ppm]
**HDA**				
**CO**			177.6	176.74
**CH_2**	2.46	2.43	45.5	44.64
**CHOH**	4.12	4.12	70.7	70.01
**CH_2**	1.55	1.53	39.4	38.55
**CH_2**	1.31	1.37	26.5	26.87
**CH_2**	1.32	1.32	31.6	30.43
**CH_2**	1.33	1.31	31.3	30.70
**CH_2**	1.33	1.30	33.9	33.00
**CH_2**	1.32	1.32	24.6	23.73
**CH_3**	0.90	0.90	15.4	14.48
**Leu-1**				
**NH**	7.66	7.60		
**CH**	4.37	4.35	55.6	55.00
**CO**			176.3	175.06
**CH_2**	1.96 / 1.67	2.01 / 1.63	43.1	42.15
**CH**	1.67	1.60	26.6	25.80
**CH_3**	0.91	0.84	21.9	21.3
**CH_3**	0.96	1.00	23.8	23.0
**Glu-2**				
**NH**	9.32	9.06		
**CH**	4.13	4.23	56.9	56.80
**CO**			177.8	176.74
**CH_2**	2.12	2.07	28.0	25.62
**CH_2**	2.49	2.51	33.1	31.07
**CO**			177.0	176.13
**Thr-3**				
**NH**	8.81	8.45		
**CH**	4.18	4.17	65.8	61.96
**CO**			177.6	172.88
**CH**	5.49	5.48	71.4	70.62
**CH_3**	1.40	1.37	19.5	18.47
**Val-4**				
**NH**	7.38	7.43		
**CH**	3.62	3.51	65.9	65.55
**CO**			174.3	174.51
**CH**	2.23	2.18	31.2	30.34
**CH_3**	0.93	0.96	20.5	19.69

**CH_3**	0.91	0.96	22.0	22.80
**Leu-5**				
**NH**	7.75	8.80		
**CH**	4.02	4.02	58.6	54.49
**CO**			175.9	175.24
**CH_2**	1.75 / 1.66	1.78 / 1.63	41.6	40.60
**CH**	1.77	1.77	26.6	25.67
**CH_3**	0.92	1.00	24.7	23.7
**CH_3**	0.91	1.00	22.3	21.3
**Ser-6**				
**NH**	8.28	8.30		
**CH**	4.51	4.50	58.5	57.70
**CO**			176.1	170.6
**CH_2OH**	3.78 / 4.03	3.94 / 3.79	63.8	63.05
**Leu-7**				
**NH**	8.44	8.41		
**CH**	4.04	3.82	55.4	54.04
**CO**			176.0	175.37
**CH_2**	1.55	2.01 / 1.68	41.3	37.86
**CH**	1.67	1.60	26.7	26.65
**CH_3**	0.96	0.90	23.7	21.3
**CH_3**	0.92	1.00	24.5	24.1
**Ser-8**				
**NH**	7.66	7.62		
**CH**	4.41	4.41	58.5	57.56
**CO**			173.9	173.72
**CH_2OH**	3.96 / 4.17	4.17 / 3.97	64.8	63.05
**Ile-9**				
**NH**	7.12	7.13		
**CH**	4.63	4.60	58.6	57.64
**CO**			170.9	170.10
**CH**	2.00	2.01	38.5	37.53
**CH_3**	0.93	0.89	17.1	16.24
**CH_2**	1.48	1.62 / 1.32	27.5	26.65
**CH_3**	0.90	0.90	13.2	12.34

Table S3. Antifungal metabolites produced by bioactive *Pseudomonas cichorii* isoated from the skin of *C. crassidigitus*. MS/MS fragmentation patterns are linked with their sequential losses of the amino acid residues.

Cyclic lipopeptides	*m/z*	MS^2 fragment ions	Amino acids residues
**Viscosin**	1125.69	994.67, 907.58, 794.50, 707.48, 594.40, 495.31, 401.24 and 266.24	Ile-Ser-Leu-Ser-Leu-Val-Thr-Glu
**Massetolide A**	1139.73	1008.65, 921.61, 808.52, 721.50, 608.41, 509.34, 409.28 and 279.52	Leu-Ser-Leu-Ser-Leu-Ile-Thr-Glu
**Massetolide F**	1126.70	995.58, 908.57, 795.47, 708.45, 597.37, 496.31, 395.24 and 265.21	Leu-Ser-Leu-Ser-Leu-Val-Thr-Glu
**Massetolide G**	1140.73	1008.65, 921.63, 808.52, 721.50, 608.42, 509.35, 408.64 and 279.52	Leu-Ser-Leu-Ser-Leu-Val-Thr-Glu
**Massetolide H**	1153.76	1022.65, 935.64, 822.55, 735.52, 622.43, 523.35, 422.30 and 292.93	Leu-Ser-Leu-Ser-Leu-Val-Thr-Glu
**Massetolide L**	1097.67	966.56, 879.59, 767.49, 679.41, 566.37, 467.30, 367.01 and 254.39	Ile-Ser-Ile-Ser-Ile-Val-Thr-Asn

Supplementary references

1. Blanco-Abad, V., Ansede-Bermejo, J., Rodriguez-Castro, A. & Martinez-Urtaza, J. Evaluation of different procedures for the optimized detection of Vibrio parahaemolyticus in mussels and environmental samples. *Int. J. Food Microbiol.* **129,** 229–236 (2009).

2. Letunic, I. & Bork, P. Interactive Tree of Life v2: Online annotation and display of phylogenetic trees made easy. *Nucleic Acids Res.* **39,** 475–478 (2011).

3. Letunic, I. & Bork, P. Interactive Tree Of Life (iTOL): an online tool for phylogenetic tree display and annotation. *Bioinformatics* **23,** 127–8 (2007).

4. Zarai, Z. *et al.* The in-vitro evaluation of antibacterial, antifungal and cytotoxic properties of *Marrubium vulgare* L. essential oil grown in Tunisia. *Lipids Health Dis.* **10,** 161 (2011).

5. Quinn, R. A. *et al.* Molecular networking as a drug discovery, drug metabolism, and precision medicine strategy. *Trends Pharmacol. Sci.* **38,** 143–154 (2017).

6. Allard, S., Allard, P.-M., Morel, I. & Gicquel, T. Application of a molecular networking approach for clinical and forensic toxicology exemplified in three cases involving 3-MeO-PCP, doxylamine, and chlormequat. *Drug Test. Anal.* (2018).

7. Wang, M. *et al.* Sharing and community curation of mass spectrometry data with Global Natural Products Social Molecular Networking. *Nat. Biotechnol.* **34,** 828–837 (2016).
